# Ablation of Discoidin Domain Receptor 1 Provokes an Osteopenic Phenotype by Regulating Osteoblast/Osteocyte Autophagy and Apoptosis

**DOI:** 10.3390/biomedicines10092173

**Published:** 2022-09-02

**Authors:** Hsin-Chiao Chou, Sung-Yen Lin, Liang-Yin Chou, Mei-Ling Ho, Shu-Chun Chuang, Tsung-Lin Cheng, Lin Kang, Yi-Shan Lin, Yan-Hsiung Wang, Chun-Wang Wei, Chung-Hwan Chen, Chau-Zen Wang

**Affiliations:** 1 Graduate Institute of Medicine, College of Medicine, Kaohsiung Medical University, Kaohsiung 80708, Taiwan; 2 Orthopaedic Research Center, Kaohsiung Medical University, Kaohsiung 80708, Taiwan; 3 Regeneration Medicine and Cell Therapy Research Center, Kaohsiung Medical University, Kaohsiung 80708, Taiwan; 4 Department of Orthopedics, Kaohsiung Medical University Hospital, Kaohsiung Medical University, Kaohsiung 80701, Taiwan; 5 Departments of Orthopedics, College of Medicine, Kaohsiung Medical University, Kaohsiung 80708, Taiwan; 6 Department of Orthopedics, Kaohsiung Municipal Ta-Tung Hospital, Kaohsiung City 80145, Taiwan; 7 Department of Physiology, College of Medicine, Kaohsiung Medical University, Kaohsiung 80708, Taiwan; 8 Department of Marine Biotechnology and Resources, National Sun Yat-sen University, Kaohsiung 80424, Taiwan; 9 Department of Obstetrics and Gynecology, National Cheng Kung University Hospital, College of Medicine, National Cheng Kung University, Tainan 70101, Taiwan; 10 School of Dentistry, College of Dental Medicine, Kaohsiung Medical University, Kaohsiung 80708, Taiwan; 11 Institute of Medical Science and Technology, National Sun Yat-Sen University, Kaohsiung 80424, Taiwan; 12 Department of Healthcare Administration and Medical Informatics, Kaohsiung Medical University, Kaohsiung 80708, Taiwan; 13 Ph.D. Program in Biomedical Engineering, College of Medicine, Kaohsiung Medical University, Kaohsiung 80708, Taiwan; 14 Graduate Institute of Animal Vaccine Technology, College of Veterinary Medicine, National Pingtung University of Science and Technology, Pingtung 912301, Taiwan; 15 Department of Medical Research, Kaohsiung Medical University Hospital, Kaohsiung 80708, Taiwan; 16 College of Professional Studies, National Pingtung University of Science and Technology, Pingtung 912301, Taiwan

**Keywords:** apoptosis, autophagy, bone loss, discoidin domain receptors 1 (DDR1), osteopenia

## Abstract

Discoidin domain receptor 1 (DDR1) is a collagen receptor that belongs to the receptor tyrosine kinase family. We have previously shown that DDR1 plays a crucial role during bone development, resulting in dwarfism and a short stature in osteoblast-specific knockout mice (OKO mice). However, the detailed pathophysiological effects of DDR1 on bone development throughout adulthood have remained unclear. This study aims to identify how DDR1 regulates osteoblast and osteocyte functions in vivo and in vitro during bone development in adulthood. The metabolic changes in bone tissues were analyzed using Micro-CT and immunohistochemistry staining (IHC) in vivo; the role of DDR1 in regulating osteoblasts was examined in MC3T3-E1 cells in vitro. The Micro-CT analysis results demonstrated that OKO mice showed a 10% reduction in bone-related parameters from 10 to 14 weeks old and a significant reduction in cortical thickness and diameter compared with flox/flox control mice (FF) mice. These results indicated that DDR1 knockout in OKO mice exhibiting significant bone loss provokes an osteopenic phenotype. The IHC staining revealed a significant decrease in osteogenesis-related genes, including RUNX2, osteocalcin, and osterix. We noted that DDR1 knockout significantly induced osteoblast/osteocyte apoptosis and markedly decreased autophagy activity in vivo. Additionally, the results of the gain- and loss-of-function of the DDR1 assay in MC3T3-E1 cells indicated that DDR1 can regulate the osteoblast differentiation through activating autophagy by regulating the phosphorylation of the mechanistic target of rapamycin (p-mTOR), light chain 3 (LC3), and beclin-1. In conclusion, our study highlights that the ablation of DDR1 results in cancellous bone loss by regulating osteoblast/osteocyte autophagy. These results suggest that DDR1 can act as a potential therapeutic target for managing cancellous bone loss.

## 1. Introduction

Discoidin domain receptors (DDRs) are receptor tyrosine kinases that recognize collagen fibrils and trigger a ligand-induced kinase activation, thereby regulating cell proliferation, differentiation, migration, and survival and playing vital roles in organ biogenesis [1]. The global DDR1 knockout mice were found to be with multiple organ dysfunctions such as lactation defects, reproductive disorders, auditory dysfunction, or kidney abnormalities [2]. These genetically deficient mice also exhibited a dwarfism-like phenotype, suggesting an important regulatory role of DDR1 in skeletal development. Further, we discovered that the cartilage-specific deletion of DDR1 delayed the endochondral ossification by modulating the terminal differentiation and apoptosis through the Ihh (Indian hedgehog)/Gli1/Gli2/ColX (type X collagen) signaling pathway [3]. Our findings indicated that DDR1 is critical for bone formation and may also be a modulating target protein for bone-related disorders.

Bone homeostasis is the process of bone formation and resorption, regulated by the balance of osteoblasts, osteoclasts, and osteocytes [4,5]. Osteocytes, the terminally differentiated stage of osteoblasts, are the longest living bone cells and compose 90% to 95% of all bone cells in adult bones, living upward of decades within their mineralized environments [6]. Osteocytes can directly control the production and function of osteoblasts and osteoclasts through gap junctions or indirectly by transmitting molecular signaling. Apoptosis is programmed cell death that modulates bone turnover by autophagically removing damaged bone cells and allowing bone cells to self-renew and maintain bone strength. The deregulation of apoptosis may contribute to the alternation in the life span of osteocytes, resulting in the pathogenesis of bone loss disease [7,8]. Numerous studies have shown that inhibiting osteocyte apoptosis can effectively reduce bone loss [9,10].

Autophagy and apoptosis are closely interconnected with bone metabolism. Enhancing the autophagy activity in osteoblasts can positively regulate skeletal cell survival [11,12], control bone cell crosstalk by decreasing cell apoptosis, and increase the mineralization capacity of osteoblasts [13]. Another study indicated that estradiol-induced autophagy activity further inhibited osteoblast apoptosis by increasing the phosphorylation of extracellular signal-regulated kinases [14]. The autophagy activator, rapamycin, restores the osteogenic differentiation and proliferation of aged bone-marrow-derived mesenchymal stem cells and reduces age-related bone loss in vivo [15]. Our previous study showed that the intraarticular injection of the DDR1 inhibitor ameliorated osteoarthritis by attenuating chondrocyte apoptosis and modulating autophagy activity in chondrocytes [16]. Accordingly, we speculated that DDR1 may be a key crosstalk factor between the levels of osteoblast/osteocyte autophagy activity and apoptosis.

DDR1 is an essential regulator of bone growth and skeletal development. Osteoblast-specific DDR1 deletion in the early developmental stage resulted in postnatal skeletal dysplasia [17]. However, the role of DDR1 on bone hemostasis in adults remained elusive. This study aimed to investigate the impact of DDR1 ablation on bone hemostasis in adulthood and further determine whether the biological alternations are controlled by modulation apoptosis and autophagy.

## 2. Materials and Methods

### 2.1. Generation of Osteoblast-Specific Knockout Mice (OKO)

We generated osteoblast-specific knockout mice (OKO mice) in [17]. First, we generated conditional Ddr1 flox/flox mice (FF), then mated them with the 4-hydroxytamoxifen (4-OHT)-dependent type I collagen-Cre recombinases (Col1al-are/ERT) mice, which were acquired from Jackson Laboratories (Bar Harbor, ME, USA). Genotyping was confirmed through polymerase chain reaction analysis with paired primers (forward: 5′-ATAGCGGCCGCTGCTGGTCTTAGCTCTGT-3′; reverse: 5′-ATAGTCGACACAGAGAGTTAAGCCAGA-3′). Our previous study results showed no difference between male and female mice. Therefore, in this study, each group of 8 female mice per genotype was analyzed at 2 weeks old. All mice were housed within a specific-pathogen-free (SPF level) environment and fed with a normal diet during the entire experimental process, with fertile conditions that exhibited an average phenotype.

### 2.2. Induction of 4-Hydroxytamoxifen (4-OHT) for Cre Recombinase Activation

The use of 4-hydroxytamoxifen (T5648, St. Louis, MO, USA) with the Cre-LoxP system followed the report of Zhong et al. in 2015 [18]. The Col-I-CreERT recombinase was activated by 4-OHT intraperitoneal injection. The injection dose had no side effects or benefits on bone and cartilage development in our previous studies [3,17]. Both FF and OKO mice were injected with 4-OHT (4 mg/day/kg) for 3 consecutive days per week at 7 to 10 weeks old and then one dose per week until they were euthanized.

### 2.3. High-Resolution MicroCT Analyses of FF and OKO Mice

High-resolution images of the distal femora using a model 1076 scanner (Skyscan; SkyScan NV, Kontich, Belgium), imaged with an X-ray tube voltage of 50 kV and a current of 200 μA, with a 0.5 mm aluminum filter and an exposure time of 800 ms. NRecon (version 1.6.1.7; SkyScan NV, Kontich, Belgium) was used to perform the reconstruction of 3D morphometric parameters after the scans. The region of interest (ROI) was evaluated on 2.5 mm of the trabecular bone starting at 0.5 mm below the growth plate. The cortical bone ROI was evaluated at 1 mm from the middle of the femur [19,20,21,22].

### 2.4. Analyses of the Histology and Immunohistochemistry Staining (IHC) in FF and OKO Mice

The mice femur was fixed in 10% buffered paraformaldehyde for 2 days, decalcified in EDTA (0.5 M EDTA, pH 7.4), embedded in paraffin, sectioned (5 μm thick), and subjected to hematoxylin and eosin (H&E) and immunochemistry (IHC) staining analyses. The IHC staining was performed by a mouse- and rabbit-specific HRP/DAB (ABC) Detection IHC kit (ab64264, Abcam, Cambridge, MA, USA) following the manufacturer’s instructions. The primary antibodies used in the OKO/FF mice were rabbit polyclonal antibodies to DDR1 (ab22719, Abcam, Cambridge, MA, USA). The primary antibodies of bone formation and osteoblast differentiation required proteins for osteogenesis-related proteins, including RUNX2 (ab23981, Abcam, Cambridge, MA, USA), osteocalcin (ARG54605, Arigo biolaboratories, Taipei, Taiwan), and osterix (ab209484, Abcam, Cambridge, MA, USA). The primary antibodies for apoptotic proteins, a frequently activated death protease, activated caspase-3 (ab2302, Abcam, Cambridge, MA, USA). The primary antibodies for autophagy-related proteins included a negative regulator of autophagy, the mammalian target of rapamycin (mTOR), phosphorylated-mTOR (Ser235/236) (4858s, Cell Signaling, Danvers, MA, USA), and autophagy-required proteins, i.e., light chain 3 (LC3) (14600-1-AP, Proteintech, Rosemont, IL, USA) and beclin-1 (11306-1-AP, Proteintech, Rosemont, IL, USA) [23,24,25,26,27,28,29,30,31]. The sections were counterstained with hematoxylin, and the immunolocalized nuclei were stained brown. All the quantitative data analyses were performed by counting the percentage of immunopositive cells in hematoxylin under a Leica-DM1750 microscope (Leica Microsystems, Wetzlar, Germany).

### 2.5. Bone Formation Rate

The dynamic histomorphometric was determined using fluorochrome markers, including calcein (Green) and alizarin red S (Rad), in 12-week-old FF and OKO mice (each group, *n* = 6). FF/OKO mice were intraperitoneally injected with 5 mg/kg calcein (Sigma-Aldrich, St. Louis, MO, USA; Merck KGaA, Darmstadt, Germany) at 8 weeks old and 20 mg/kg alizarin red S (Sigma-Aldrich; Merck KGaA) at 12 weeks old. The parameters related to bone formation were analyzed by the double-labeled marker of bone surfaces, and the inter-label width in the cancellous bone of the distal femur metaphysis was measured. The femur is the single bone of the thigh, where bone formation changes are easily observed. A mineralized over bone surface (MS/BS, %) was the percentage of bone surface between two fluorochrome marker labels, reflecting active mineralization. The mineral apposition rate (MAR) was the linear rate of new bone deposition (μm/day). The daily bone formation rate was calculated by multiplying the mineralizing surface and the mineral apposition rate.

### 2.6. Staining Osteocyte Apoptosis by Terminal Deoxynucleotidyl Transferase dUTP Nick End Labeling (TUNEL) Assay

TUNEL staining was performed to detect osteocyte apoptosis (12156792910, Roche, Basel, Switzerland). The percentages of TUNEL-positive cells in osteocytes relative to 4′,6-diamidino-2-phenylindole (DAPI)-stained cells were calculated, and an analysis was conducted using a Leica immunofluorescence system (Leica Microsystems, Wetzlar, Germany). Photographs from three independent experiments were taken for each experimental group [25,32].

### 2.7. Western Blot Analysis

Western blot analysis was used to detect osteogenesis markers and autophagy-related proteins in cultured mouse osteoblast MC3T3-E1 cells. Cell lysates were harvested on ice in a RIPA Lysis buffer (89901 Thermo Fisher Scientific Inc., Waltham, MA, USA). Cell lysates were mixed with a 5× loading buffer and boiled in water at 100 °C for 10 min. A total of 50 μg of protein was isolated using 10% SDS-PAGE for 2 h and transferred to polyvinylidene difluoride membranes (PVDF; Millipore, Burlington, MA, USA). This was followed by blocking with 5% non-fat milk and incubation overnight at 4 °C with the following primary antibody: DDR1 (ab22719, Abcam, Cambridge, MA, USA). The primary antibodies of bone formation and osteoblast differentiation required proteins for osteogenesis-related proteins, including RUNX2 (ab23981, Abcam, Cambridge, MA, USA), Col-I (ab34710, Abcam, Cambridge, MA, USA), and osterix (ab209484, Abcam, Cambridge, MA, USA). The primary antibody for apoptotic proteins, a frequently activated death protease, was activated caspase-3 (ab2302, Abcam, Cambridge, MA, USA). The primary antibodies for autophagy-related proteins included a negative regulator of autophagy, the mammalian target of rapamycin (mTOR), i.e., phosphorylated-mTOR (Ser235/236) (4858s, Cell Signaling, Danvers, MA, USA), Total-mTOR (2217S, Cell Signaling, Danvers, MA, USA), and autophagy-required proteins, i.e., light chain 3 (LC3) (14600-1-AP, Proteintech, Rosemont, IL, USA) and beclin-1 (11306-1-AP, Proteintech, Rosemont, IL, USA) [27,33,34]. The membranes were washed in PBS for 30 min (10 min/time) and incubated with HRP-labeled goat anti-mouse and IgG/HRP-labeled goat anti-rabbit IgG for 1 h at room temperature. The blots were detected by enhanced chemiluminescence analysis (ECL system; GE Healthcare, Piscataway, NJ, USA).

### 2.8. Statistical Analysis

All data are presented as mean ± SEM. The results were analyzed using one-way ANOVA, and multiple comparisons were conducted by Tukey’s HSD using GraphPad Prism (version 5.0). The statistical significance was considered *p* < 0.05.

## 3. Results

### 3.1. OKO Mice Showed a Significant Decrease in Osteoblast/Osteocyte DDR1 Level in Femoral Trabecular and Cortical Bones

**Figure 1:**
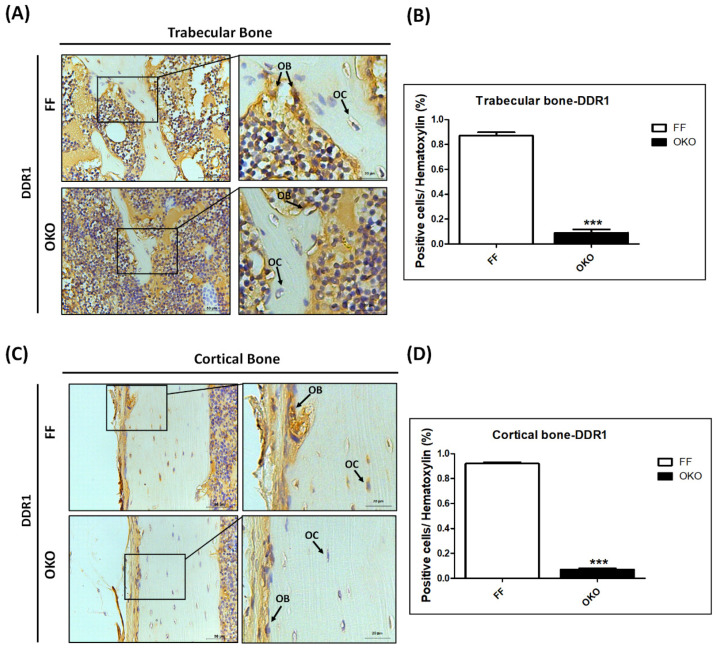
**Ablation of DDR1 in osteoblasts/osteocytes showed a significant DDR1 protein level decrease in femoral trabecular and cortical bones.** (**A**) The representative micrographs of IHC staining of DDR1 in trabecular bone. (**B**) Quantitative results of DDR1 in trabecular bone. (**C**) The representative micrographs of IHC staining of DDR1 in cortical bone. (**D**) Quantitative results of DDR1 in cortical bone; OB: osteoblasts, OC: osteocytes. The representative Micro-CT images are shown with scale bars of 50 μm, and the zoomed-in images are 20 μm. The positive immunolocalizations are stained dark brown. In the quantitative analysis, each bar represents the mean ± SE of eight samples in each group (*** *p* < 0.001 versus FF).

We performed IHC staining to evaluate the deletion rate of DDR1 in osteoblasts/osteocytes in OKO mice. The results showed that OKO mice exhibited a significant decrease in DDR1-positive cells, both in osteoblasts and osteocytes, in trabecular (Figure 1A,B) and cortical bones (Figure 1C,D) compared with FF mice.

### 3.2. Deletion of DDR1 in Osteoblasts Induced Cancellous Bone Loss and Displayed Decreased Cortical Bone Thickness and Diameter

**Figure 2:**
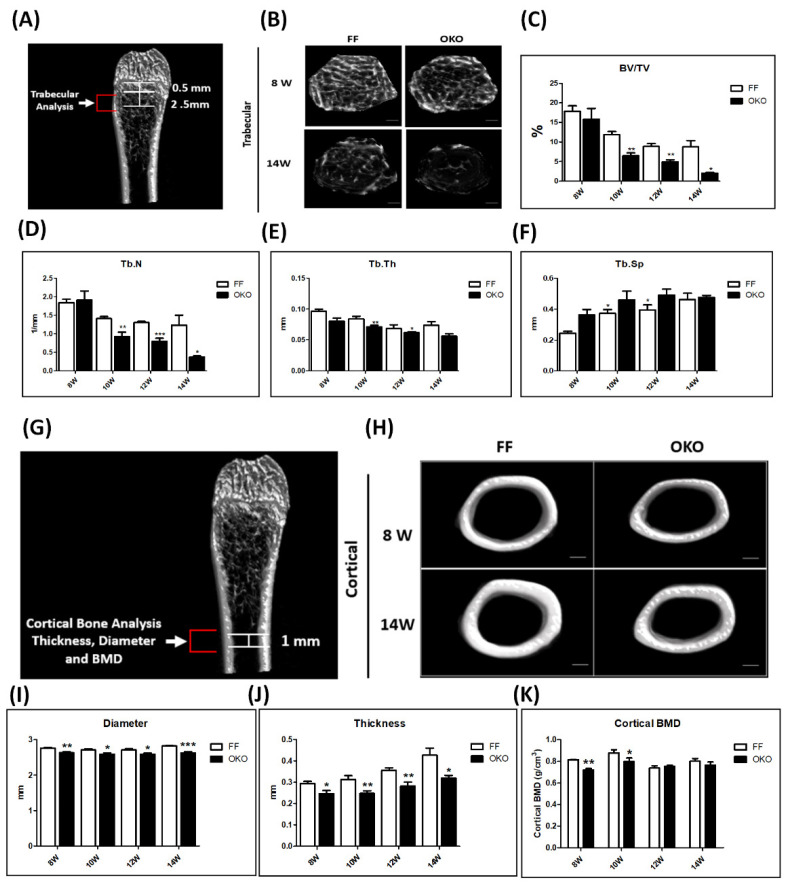
**Deletion of DDR1 in osteoblasts/osteocytes decreased cancellous bone volume in femur trabecular bone and showed a thinning cortical bone in the femur.** (**A**) The region of the trabecular bone cross section includes evaluating 2.5 mm of the trabecular bone starting at 0.5 mm below the growth plate. The representative Micro-CT images of the distal femoral of FF/OKO mice. (**B**) The femur trabecular bone in the 3D structure of FF and OKO mice at 8 and 14 weeks old. (**C**) The quantitative results of the bone/tissue volume (BV/TV). (**D**) Trabecular number (Tb.N). (**E**) Trabecular thickness (Tb.Th) and (**F**) trabecular separation (Tb.Sp) in FF/OKO mice between 8 and 14 weeks old. (**G**) The 1-mm selected region of the cortical bone cross section was analyzed in the middle of the femur. The representative Micro-CT images of the diaphysis of the femur of FF/OKO mice are shown. (**H**) The transverse section view of the femur shaft at 8 and 14 weeks old. (**I**) Quantitation of the thickness (**J**) diameter and the (**K**) bone mineral density (BMD). The representative Micro-CT images are shown with scale bars of 50 μm. In the quantitative analysis, each bar represents the mean ± SE of eight samples in each group (* *p* < 0.05 versus FF; ** *p* < 0.01 versus FF; *** *p* < 0.001 versus FF).

To determine whether DDR1 deletion in osteoblasts/osteocytes affected cancellous bone homeostasis in young adults, we examined the skeletal phenotypes of 8- to 14-week-old OKO and FF control mice. To characterize the morphology of the cortex bone and the microarchitecture of the trabecular bone, distal femurs were scanned using a high-resolution Micro-CT scanner (Figure 2A). The representative images of Micro-CT examinations at 8 and 14 weeks old are shown in Figure 2B. The results showed that the cancellous bone decreased by 10% in bone/tissue volume (BV/TV) between 10 and 12 weeks old. The trabecular number (Tb.N) and thickness (Tb.Th) significantly decreased and the trabecular separation (Tb.Sp) significantly increased between 10 and 12 weeks old (Figure 2C–F). We also examined the cortical bone thickness and diameter with a Micro-CT analysis (Figure 2G,H). The results showed a significant reduction in the cortical outer thickness and diameter (Figure 2I–J), but no influence on the cortical inner-thickness (data not shown) in OKO mice compared with those in FF control mice (*p* < 0.05). The results also showed a significant decrease in the cortex bone mineral density (BMD) from 8 to 10 weeks old in OKO mice (Figure 2K). These results indicated that DDR1 knockout in osteoblasts/osteocytes caused decreased cancellous and cortical bone volume in young adulthood mice, contributing to presentations of the osteopenia phenotype.

### 3.3. Deletion of DDR1 in Osteoblasts Decreased Bone Formation in OKO Mice

**Figure 3:**
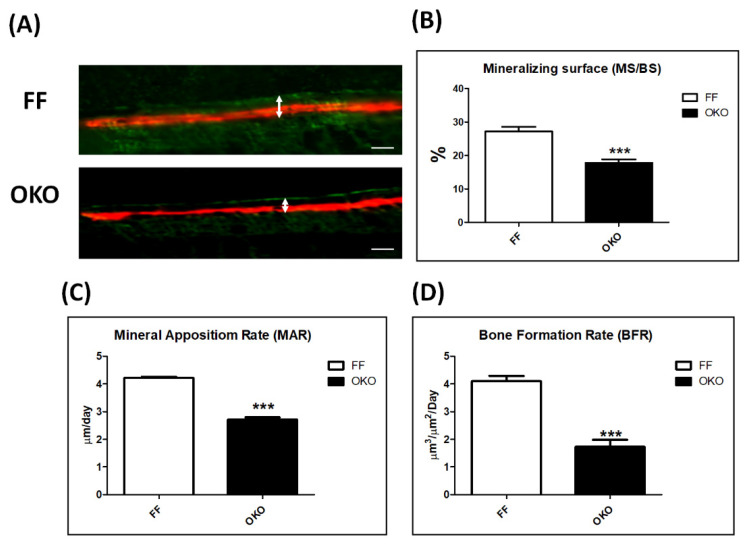
**Decreased bone formation rate in the femurs of DDR1 knockout mice.** (**A**) Representative calcein and alizarin red S labeling images demonstrating cortical bone formation in the cortical endosteum of the distal femora of 12-week-old FF/OKO mice. The white arrow points to the label of the distance between calcein (Green) and alizarin red S (Red) dyes in a time interval of 28 days. Quantitation of dynamic histomorphometry parameters of periosteum, including (**B**) the mineralized over bone surface (MS/BS), (**C**) the mineral apposition rate (MAR), and (**D**) the bone formation rate (BFR) in the distal femur metaphysis of FF and OKO mice. The representative images of fluorochrome marker labels are shown with scale bars of 50 μm. In the quantitative analysis, each bar represents the mean ± SE of six samples in each group (*** *p* < 0.001 versus FF mice).

To assess the effects of DDR1 in bone formation, we next investigated DDR1 knockout in osteoblasts/osteocytes in OKO mice compared with those in FF control mice with bone labeling and dynamic histomorphometric analyses. The representative images presented the distal femurs in OKO mice compared with those in FF mice, and the thickness of the fluorochrome-marker-labeled cortex bone showed significant attenuation in the mineralizing surface and mineral apposition rate (Figure 3A). The results exhibited a significant decrease in the mineralized over bone surface (MS/BS, %) (Figure 3B), mineral apposition rate (MAR) (Figure 3C), and bone formation rate (BFR) (Figure 3D) in DDR1-deficient mice. These findings indicated that DDR1 knockout in osteoblasts/osteocytes significantly suppressed the functional capacity of the average osteoblasts, causing an approximately 20% reduction in bone formation.

### 3.4. OKO Mice Showed a Significant Decrease in the Expression of Osteogenesis-Related Proteins

**Figure 4:**
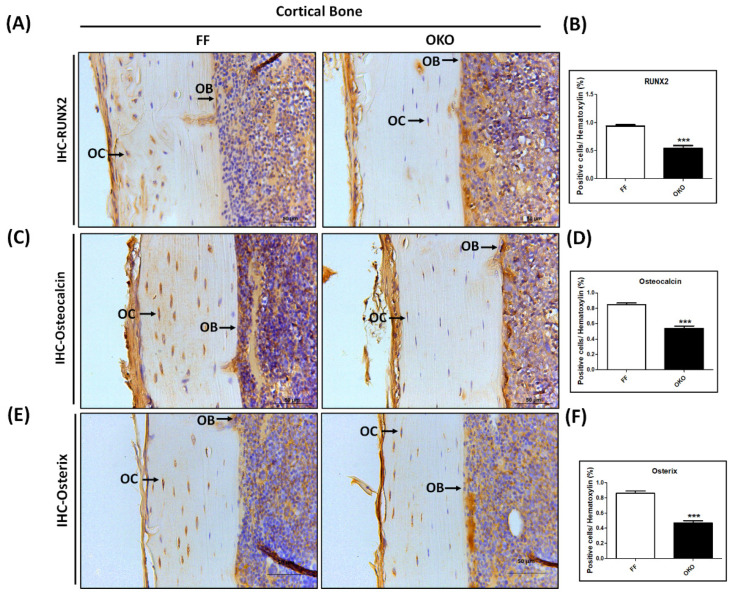
**DDR1 deficiency significantly reduced osteogenesis-related protein expression.** (**A**) The representative micrographs of immunolocalized osteogenesis-related protein RUNX2. (**B**) Quantitative results of RUNX2. (**C**) The representative micrographs of immunolocalized osteogenesis-related protein osteocalcin. (**D**) Quantitative results of osteocalcin. (**E**) The representative micrographs of immunolocalized osteogenesis-related protein osterix. (**F**) Quantitative results of osterix. All results were present in the cortical bone of the femur in 12-week-old FF and OKO mice. The positive immunolocalizations are stained dark brown. A black arrow indicates positive cells (OB + OC); OB: osteoblasts, OC: osteocytes. Magnifications of 400× are shown, with scale bars of 50 μm. Each bar represents the mean ± SE of six samples in each group (*** *p* < 0.001 versus FF mice).

To investigate whether DDR1 knockout in osteoblasts/osteocytes has a significant influence on the level of osteogenesis-related proteins, we performed IHC staining in OKO and FF mice. The representative images of IHC staining of osteogenesis-related protein markers in 12-week-old OKO mice are shown in Figure 4A,C,E, and the results of the quantitative analysis are shown in Figure 4B,D,F. The immunostained osteogenesis-related proteins, in terms of RUNX2, osteocalcin, and osterix, decreased after DDR1 knockout in the osteoblast in OKO mice. These results indicated that DDR1 knockout in osteoblasts/osteocytes significantly decreased the total bone mass when the level of osteogenesis-related proteins in OKO mice decreased compared with those in FF mice.

### 3.5. DDR1 Knockout Increased Osteocyte Apoptosis in 12-Week-Old OKO Mice

**Figure 5:**
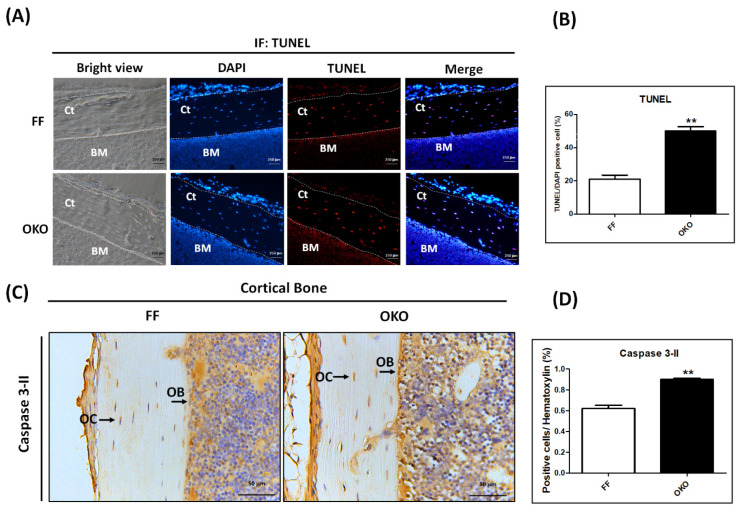
**Knockout of DDR1 in OKO mice significantly increased apoptotic osteoblasts/osteocytes.** (**A**) TUNEL staining in femoral cortical bone of FF and OKO mice. The magnification is 100×, and the scale bar is 250 μm. Red: TUNEL-positive cells; blue: DAPI-stained cells. (**B**) Quantification of TUNEL-positive cells in the area beneath the cortical bone. (**C**) The representative micrographs of IHC of activated caspase-3 (caspase 3-II). (**D**) Quantitative analysis of the IHC staining of activated caspase-3. The positive immunolocalizations are stained dark brown. A black arrow indicates positive cells (OB + OC); OB: osteoblasts, OC: osteocytes. Magnifications of 400× are shown, with scale bars of 50 μm. Each bar represents the mean ± SE of six samples in each group (** *p* < 0.01 versus FF mice).

To determine the relevance of osteoblast/osteocyte apoptosis after the deletion of DDR1, we examined the femurs of OKO/FF mice with TUNEL staining. The results showed that the deletion of DDR1 in osteoblasts/osteocytes revealed more osteoblast/osteocyte apoptosis in OKO mice (Figure 5A). The apoptotic rate in OKO mice (50 ± 2.65%) significantly increased compared to that in the FF mice (21 ± 2.45%, *p* < 0.001) (Figure 5B). The expression of activated caspase-3, a crucial mediator of apoptosis, also significantly increased after DDR1 knockout (Figure 5C,D).

### 3.6. DDR1 Knockout Significantly Increased Autophagy-Related Markers—mTOR, LC3, and Beclin-1 Expressions—In OKO Mice

**Figure 6:**
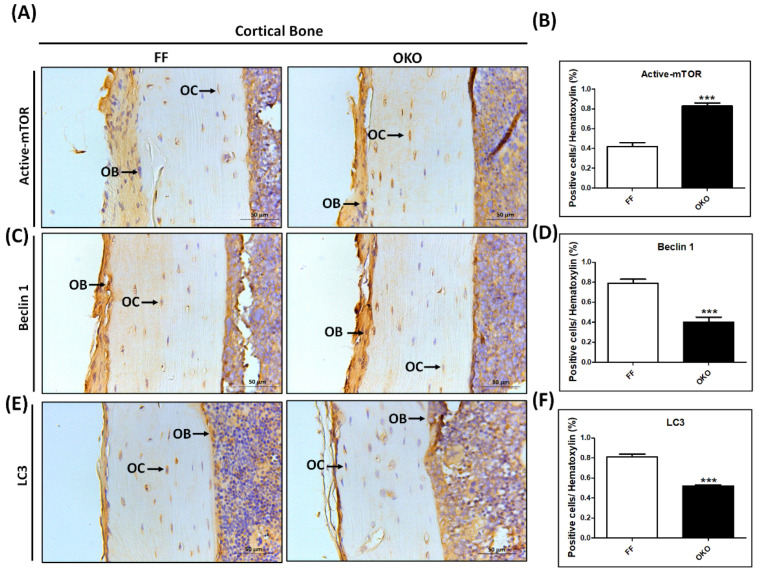
**DDR1 knockout significantly decreased autophagy-related protein levels in OKO mice.** (**A**) The representative IHC of the phosphorylation of the mechanistic target of rapamycin (phospho-mTOR) in FF and OKO mice. (**B**) Quantitative analysis of the IHC staining of phospho-mTOR. (**C**) The representative IHC of beclin-1. (**D**) Quantitative analysis of IHC of beclin-1. (**E**) The representative IHC of light chain 3 (LC3). (**F**) Quantitative analysis of IHC of LC3. The positive immunolocalizations are stained dark brown. A black arrow indicates positive cells (OB + OC); OB: osteoblasts, OC: osteocytes. Magnifications of 400× are shown, with scale bars of 50 μm. Each bar represents the mean ± SE of six samples in each group (*** *p* < 0.001 versus FF mice).

Autophagy is a master regulator of cellular metabolism. To determine whether DDR1 knockout in osteoblasts/osteocytes results in reduced autophagy, we used IHC staining to examine the expression of autophagy-related markers, including mTOR, LC3, and beclin-1 in the femur bones of FF/OKO mice (Figure 6A,C,E). We found a significant increase in the level of phosphorylated mTOR in the OKO mice compared with that in the FF control mice (Figure 6B). Additionally, the expression levels of LC3 and beclin-1 in OKO mice were significantly lower than that in the FF control mice (Figure 6D,F). These results indicated a decrease in osteoblast/osteocyte autophagy after DDR1 knockout in OKO mice.

### 3.7. DDR1 Is Required for Osteogenesis and Autophagy Induction in MC3T3-E1 Cells

**Figure 7:**
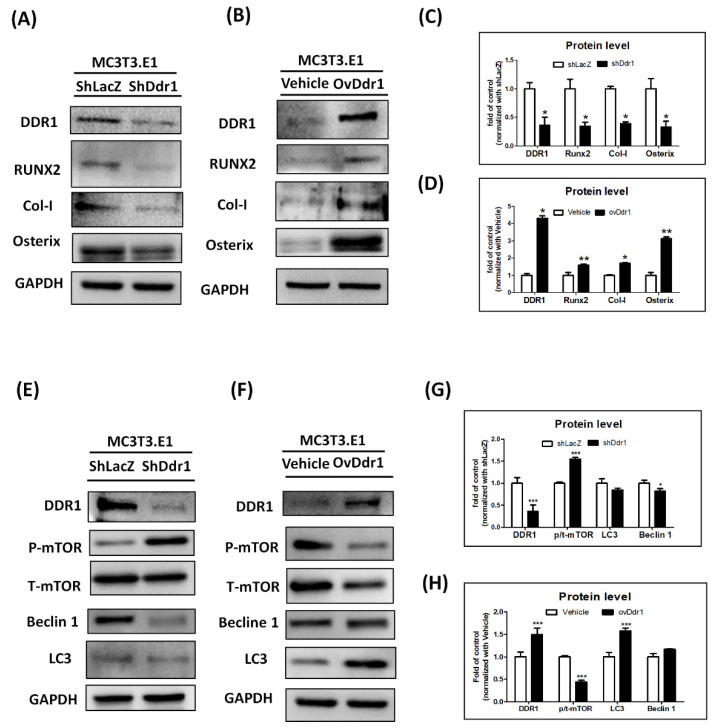
**DDR1 enhanced osteogenesis and decreased autophagy in MC3T3-E1 cells.** (**A**,**B**) Western blotting against DDR1, RUNX2, Col-I, osterix, and GAPDH in MC3T3-E1 cells infected with shLacZ/shDDR1 and Vehicle/ovDDR1. (**C**,**D**) Quantitation results of DDR1, RUNX2, Col-I, and osterix for shDDR1 compared with those of shLacZ and ovDDR1 compared with those of the vehicle. (**E**,**F**) Western blotting against DDR1, p-mTOR, t-mTOR, beclin-1, and LC3 in MC3T3-E1 cells infected with shLacZ/shDDR1 and Vehicle/ovDDR1. (**G**,**H**) Quantitation of the results for shDDR1 compared with those for shLacZ and ovDDR1 compared with those of the vehicle. This experiment was performed in quadruplicate and repeated three times with similar results. ShLacZ/Vehicle: the transduction by empty lentivirus; RUNX2: runt-related transcription factor 2; Col-I: collagen I; Osx: osterix; GAPDH: glyceraldehyde 3-phosphate dehydrogenase; p-mTOR: phosphorylation of the mechanistic target of rapamycin; t-mTOR: total of the mechanistic target of rapamycin; LC3: light chain 3 (* *p* < 0.05 versus shLacZ or Vehicle, ** *p* < 0.01 versus shLacZ or Vehicle, *** *p* < 0.001 versus shLacZ or Vehicle).

To confirm the influence of DDR1 in autophagy, we used gain- and loss-of-function assays by lentiviral-mediated overexpression (OvDDR1)/knockdown (ShDDR1) of DDR1 in preosteoblast MC3T3-E1 cells. Firstly, we found that DDR1 increased osteogenesis-related markers in the OvDDR1 group as assessed with Western blot. The result showed that the expressions of RUNX2, Col-I, and osterix decreased in MC3T3-E1 cells with DDR1 knockdown (Figure 7A,C) and increased in MC3T3-E1 cells overexpressing DDR1 (Figure 7B,D). In terms of the effect of DDR1 on autophagy, we found that shDDR1 suppressed autophagy activity with alterations in the related markers, including the phosphorylation of p-mTOR, LC3, and beclin-1 in MC3T3-E1 cells (Figure 7E,G). On the contrary, the autophagy capability was upregulated after an overexpression of DDR1 (Figure 7F,H). Our results suggested that DDR1 knockout in osteoblasts resulted in a reduction of osteogenesis, accompanied by a decrease in autophagy activity.

## 4. Discussion

This study was the first to show, by regulating the programmed cell death of osteoblasts/osteocytes in vivo and in vitro, that DDR1 is an important regulatory factor in bone formation during adulthood. We demonstrated that DDR1 knockout in osteoblasts/osteocytes exhibited a lower bone volume in the trabecular bone, a lower thickness, and a shorter diameter in the cortical bone compared with those in the control group. The osteoblast/osteocyte apoptosis was enhanced, and the autophagy activity was suppressed after DDR1 deletion, suggesting the regulatory role of DDR1 during the programmed cell death of osteoblasts/osteocytes. In addition, the in vitro study showed that the osteogenesis- and autophagy-related proteins both increased after DDR1 overexpression and decreased after the silencing of DDR1 in preosteoblast MC3T3-E1 cells. These findings indicated that DDR1 may regulate osteogenesis by modulating the autophagy activity of osteoblasts/osteocytes.

The effects of DDR1 inhibition on osteoblasts and osteoclasts have been investigated from studies on DDR1 inhibitors. It has been reported that DDR1 inhibitors, including imatinib and nilotinib, may have a positive effect on bone metabolism through potently inhibited osteoclastogenesis, mediated by stromal-cell-dependent mechanisms or direct effects on osteoclast precursors [35]. The results of a clinical trial also showed a low to normal level of bone markers after treatment with nilotinib, despite secondary hyperparathyroidism, further revealing the antiresorptive effect of DDR1 inhibition [36]. Though previous studies have reported similar findings on osteoclasts, the effects on osteoblasts are diverse. DDR1 inhibitors potently activated osteoblast differentiation and inhibited osteoblast proliferation through inhibiting platelet-derived growth factor receptor signaling [37]. A low concentration of imatinib (0.1–1.0 μM) enhanced differentiation and mineralization in both primary rat osteoblastic cells and MC3T3-E1 cells, but 1.0 μM imatinib significantly increased osteoblast apoptosis and 50% more apoptosis at a higher concentration (5 μM) [35]. By contrast, nilotinib (0.1–0.5 μM) reduced osteoblast differentiation and mineralization [36]. The diverse effects of DDR1 inhibitors on osteoblasts suggests that osteoblastic differentiation varies with the maturation stage of cells. It was reported that imatinib promoted osteoblastic differentiation of human mesenchymal stem cells in the early stage but inhibited osteoblastic differentiation and mineralization in the late stage by reducing the expression of RUNX2, Col1, and osterix [38].

Apoptosis is essential for the balance between bone formation and resorption [39]. Dysregulated apoptosis of osteoblasts or osteocytes accounts for bone-loss-related diseases such as osteoporosis or osteonecrosis. It was reported that parathyroid hormones (PTHs) increased bone formation by reducing osteoblast apoptosis and could reverse corticosteroid-induced osteoporosis [40]. Further, catechin reduced osteoblast apoptosis contributing to a positive effect on osteogenesis [41]. In this study, we found that OKO mice revealed increased osteoblast/osteocyte apoptosis in terms of TUNEL and IHC staining during adulthood compared with the FF mice. The IHC staining of OKO mice also exhibited more osteocyte lacuna compared with that of FF mice. The increased number of osteocyte lacuna indicated an increased clearance of osteocyte material from the sites of death cells. These results supported the hypothesis that DDR1 is essential for osteoblast/osteocyte apoptosis during bone formation.

We previously reported that the osteoblast-specific deletion of DDR1 during the developmental stage reduced the osteoblast differentiation and exhibited skeletal dysplasia [17]. In the embryonic stage, endochondral ossification was gradually replaced by bone, the primary type of biological process for developing the skeleton [42]. The skeletal mass during adulthood is controlled by the coupling signals between osteoblasts and osteoclasts, which regulate the balance of bone formation and resorption [43]. Continuing our previous work on embryonic bone development, we further investigated the regulatory mechanism of DDR1 in bone formation during adulthood. We found that the increased apoptosis of osteoblasts/osteocytes was significantly coupled with reduced autophagy activity after DDR1 deletion in osteoblasts, contributing to a reduction of bone formation during adulthood. The formation of the cortical bone is more related to the osteoblasts in the periosteum, with few roles for osteoclasts [44,45]. Therefore, we also noted a significant decrease in the thickness of the cortical bone after DDR1 deletion in osteoblasts/osteocytes.

The crosstalk between apoptosis and autophagy is important in cellular metabolic survival and cellular housekeeping mechanisms [46,47,48]. It has been reported that modulating the autophagy of osteoblasts could avoid oxidative damage and allow cells to continue normal function through the endoplasmic reticulum stress pathway [49]. Li et al. showed that the autophagy inducer (rapamycin) significantly increased the autophagy activity and attenuated the oxidative-stress-mediated apoptosis of osteoblasts, and the autophagy inhibitor (3-methyladenine) reduced autophagy and enhanced hydrogen-peroxide-induced oxidative stress and apoptosis [50]. The autophagy of osteoblasts is positively involved in tissue mineralization and bone homeostasis. The deletion of autophagy-essential genes in osteoblasts increases oxidative stress and further reduces the mineralization capacity. Our study also found that the expression of autophagy genes was positively correlated with the expression of osteogenic genes, and this change was regulated by the expression of DDR1 in MC3T3-E1 cells. In our in vivo study, DDR1 deletion significantly reduced the osteoblast/osteocyte autophagy levels, resulting in more osteoblast/osteocyte apoptosis compared with that in FF control mice. We suggest that DDR1 could maintain autophagy activity and regulate osteoblast/osteocyte apoptosis, which also contributes to the achievement of normal bone homeostasis. However, the detailed mechanisms of DDR1 in regulating autophagic activity and the crosstalk in decreasing osteoblast/osteocyte apoptosis require further studies.

## 5. Conclusions

Our results demonstrated that DDR1 knockout in osteoblasts/osteocytes decreased bone formation in adult OKO mice. These phenomena, by decreasing autophagy both in vitro and in vivo, were associated with an increased apoptosis in bone-forming cells. We demonstrated that DDR1 in osteoblasts/osteocytes played a regulatory role in the balance between osteoblast/osteocyte autophagy activity and apoptosis, and it also contributed to maintaining bone formation. These findings suggested that DDR1 is an important regulator in bone formation and could be a potential therapeutic target for bone-loss disease.

## Data Availability

Data is contained within the article.

## References

[1] Leitinger B. (2003). Molecular analysis of collagen binding by the human discoidin domain receptors, DDR1 and DDR2. Identification of collagen binding sites in DDR2. J. Biol. Chem..

[2] Vogel W.F., Aszódi A., Alves F., Pawson T. (2001). Discoidin domain receptor 1 tyrosine kinase has an essential role in mammary gland development. Mol. Cell. Biol..

[3] Chou L.Y., Chen C.H., Lin Y.H., Chuang S.C., Chou H.C., Lin S.Y., Fu Y.C., Chang J.K., Ho M.L., Wang C.Z. (2020). Discoidin domain receptor 1 regulates endochondral ossification through terminal differentiation of chondrocytes. Off. Publ. Fed. Am. Soc. Exp. Biol..

[4] Harada S.-i., Rodan G.A. (2003). Control of osteoblast function and regulation of bone mass. Nature.

[5] Komori T. (2006). Regulation of osteoblast differentiation by transcription factors. J. Cell. Biochem..

[6] Tu X., Delgado-Calle J., Condon K.W., Maycas M., Zhang H., Carlesso N., Taketo M.M., Burr D.B., Plotkin L.I., Bellido T. (2015). Osteocytes mediate the anabolic actions of canonical Wnt/β-catenin signaling in bone. Proc. Natl. Acad. Sci. USA.

[7] Lirani-Galvão A.P., Chavassieux P., Portero-Muzy N., Bergamaschi C.T., Silva O.L., Carvalho A.B., Lazaretti-Castro M., Delmas P.D. (2009). Low-intensity electrical stimulation counteracts the effects of ovariectomy on bone tissue of rats: Effects on bone microarchitecture, viability of osteocytes, and nitric oxide expression. Calcif. Tissue Int..

[8] Ru J.Y., Wang Y.F. (2020). Osteocyte apoptosis: The roles and key molecular mechanisms in resorption-related bone diseases. Cell Death Dis..

[9] Chan G.K., Duque G. (2002). Age-related bone loss: Old bone, new facts. Gerontology.

[10] Liang J., Chen C., Liu H., Liu X., Li Z., Hu J., Zhao H. (2018). Gossypol promotes bone formation in ovariectomy-induced osteoporosis through regulating cell apoptosis. BioMed Res. Int..

[11] Pierrefite-Carle V., Santucci-Darmanin S., Breuil V., Camuzard O., Carle G.F. (2015). Autophagy in bone: Self-eating to stay in balance. Ageing Res. Rev..

[12] Ran D., Ma Y., Liu W., Luo T., Zheng J., Wang D., Song R., Zhao H., Zou H., Gu J. (2020). TGF-β-activated kinase 1 (TAK1) mediates cadmium-induced autophagy in osteoblasts via the AMPK/mTORC1/ULK1 pathway. Toxicology.

[13] Yin X., Zhou C., Li J., Liu R., Shi B., Yuan Q., Zou S. (2019). Autophagy in bone homeostasis and the onset of osteoporosis. Bone Res..

[14] Yang Y.H., Chen K., Li B., Chen J.W., Zheng X.F., Wang Y.R., Jiang S.D., Jiang L.S. (2013). Estradiol inhibits osteoblast apoptosis via promotion of autophagy through the ER-ERK-mTOR pathway. Apoptosis Int. J. Program. Cell Death.

[15] Ma Y., Qi M., An Y., Zhang L., Yang R., Doro D.H., Liu W., Jin Y. (2018). Autophagy controls mesenchymal stem cell properties and senescence during bone aging. Aging Cell.

[16] Chou H.C., Chen C.H., Chou L.Y., Cheng T.L., Kang L., Chuang S.C., Lin Y.S., Ho M.L., Wang Y.H., Lin S.Y. (2020). Discoidin domain receptors 1 inhibition alleviates osteoarthritis via enhancing autophagy. Int. J. Mol. Sci..

[17] Chou L.Y., Chen C.H., Chuang S.C., Cheng T.L., Lin Y.H., Chou H.C., Fu Y.C., Wang Y.H., Wang C.Z. (2020). Discoidin domain receptor 1 regulates Runx2 during osteogenesis of osteoblasts and promotes bone ossification via phosphorylation of p38. Int. J. Mol. Sci..

[18] Zhong Z.A., Sun W., Chen H., Zhang H., Lay Y.E., Lane N.E., Yao W. (2015). Optimizing tamoxifen-inducible Cre/loxp system to reduce tamoxifen effect on bone turnover in long bones of young mice. Bone.

[19] Chen C.H., Kang L., Lin R.W., Fu Y.C., Lin Y.S., Chang J.K., Chen H.T., Chen C.H., Lin S.Y., Wang G.J. (2013). (-)-Epigallocatechin-3-gallate improves bone microarchitecture in ovariectomized rats. Menopause.

[20] Chen C.H., Kang L., Lo H.C., Hsu T.H., Lin F.Y., Lin Y.S., Wang Z.J., Chen S.T., Shen C.L. (2015). Polysaccharides of trametes versicolor improve bone properties in diabetic rats. J. Agric. Food Chem..

[21] Chen C.H., Lai C.H., Hong Y.K., Lu J.M., Lin S.Y., Lee T.C., Chang L.Y., Ho M.L., Conway E.M., Wu H.L. (2020). Thrombomodulin functional domains support osteoblast differentiation and bone healing in diabetes in mice. J. Bone Miner. Res..

[22] Lin S.Y., Kang L., Chen J.C., Wang C.Z., Huang H.H., Lee M.J., Cheng T.L., Chang C.F., Lin Y.S., Chen C.H. (2019). (−)-Epigallocatechin-3-gallate (EGCG) enhances healing of femoral bone defect. Phytomedicine.

[23] Huang H.T., Cheng T.L., Yang C.D., Chang C.F., Ho C.J., Chuang S.C., Li J.Y., Huang S.H., Lin Y.S., Shen H.Y. (2021). Intra-articular injection of (-)-Epigallocatechin 3-Gallate (EGCG) ameliorates cartilage degeneration in guinea pigs with spontaneous osteoarthritis. Antioxidants.

[24] Huang H.T., Cheng T.L., Ho C.J., Huang H.H., Lu C.C., Chuang S.C., Li J.Y., Lee T.C., Chen S.T., Lin Y.S. (2021). Intra-Articular injection of (−)-Epigallocatechin 3-Gallate to attenuate articular cartilage degeneration by enhancing autophagy in a post-traumatic osteoarthritis rat model. Antioxidants.

[25] Chen C.H., Ho M.L., Chang L.H., Kang L., Lin Y.S., Lin S.Y., Wu S.C., Chang J.K. (2018). Parathyroid hormone-(1-34) ameliorated knee osteoarthritis in rats via autophagy. J. Appl. Physiol..

[26] Chang L.H., Wu S.C., Chen C.H., Wang G.J., Chang J.K., Ho M.L. (2016). Parathyroid hormone 1-34 reduces dexamethasone-induced terminal differentiation in human articular chondrocytes. Toxicology.

[27] Lin S.Y., Kan J.Y., Lu C.C., Huang H.H., Cheng T.L., Huang H.T., Ho C.J., Lee T.C., Chuang S.C., Lin Y.S. (2020). Green tea catechin (-)-Epigallocatechin-3-Gallate (EGCG) facilitates fracture healing. Biomolecules.

[28] Lin S.Y., Kang L., Wang C.Z., Huang H.H., Cheng T.L., Huang H.T., Lee M.J., Lin Y.S., Ho M.L., Wang G.J. (2018). (−)-Epigallocatechin-3-Gallate (EGCG) enhances osteogenic differentiation of human bone marrow mesenchymal stem cells. Molecules.

[29] Chou Y.S., Chuang S.C., Chen C.H., Ho M.L., Chang J.K. (2021). G-protein-coupled estrogen receptor-1 positively regulates the growth plate chondrocyte proliferation in female pubertal mice. Front. Cell Dev. Biol.

[30] Cheng T.L., Chen C.H., Wu M.H., Lai C.H., Lee K.H., Lin S.H., Shiau A.L., Wu C.L., Kang L. (2021). Upregulation of fibrinogen-like 1 expression contributes to reducing the progression of preeclampsia. Front. Cell. Dev. Biol..

[31] Chuang S.C., Chen C.H., Chou Y.S., Ho M.L., Chang J.K. (2020). G protein-coupled estrogen receptor mediates cell proliferation through the cAMP/PKA/CREB pathway in murine bone marrow mesenchymal stem cells. Int. J. Mol. Sci..

[32] Chen C.H., Cheng T.L., Chang C.F., Huang H.T., Lin S.Y., Wu M.H., Kang L. (2021). Raloxifene ameliorates glucosamine-induced insulin resistance in ovariectomized rats. Biomedicines.

[33] Yeh C.H., Chang J.K., Ho M.L., Chen C.H., Wang G.J. (2009). Different differentiation of stroma cells from patients with osteonecrosis: A pilot study. Clin. Orthop. Relat. Res..

[34] Chen C.H., Kuo S.M., Tien Y.C., Shen P.C., Kuo Y.W., Huang H.H. (2020). Steady augmentation of anti-osteoarthritic actions of rapamycin by liposome-encapsulation in collaboration with low-intensity pulsed ultrasound. Int. J. Nanomed..

[35] O’Sullivan S., Naot D., Callon K., Porteous F., Horne A., Wattie D., Watson M., Cornish J., Browett P., Grey A. (2007). Imatinib promotes osteoblast differentiation by inhibiting PDGFR signaling and inhibits osteoclastogenesis by both direct and stromal cell-dependent mechanisms. J. Bone Miner. Res. Off. J. Am. Soc. Bone Miner. Res..

[36] O’Sullivan S., Lin J.M., Watson M., Callon K., Tong P.C., Naot D., Horne A., Aati O., Porteous F., Gamble G. (2011). The skeletal effects of the tyrosine kinase inhibitor nilotinib. Bone.

[37] Tibullo D., Giallongo C., La Cava P., Berretta S., Stagno F., Chiarenza A., Conticello C., Palumbo G.A., Di Raimondo F. (2009). Effects of imatinib mesylate in osteoblastogenesis. Exp. Hematol..

[38] Jönsson S., Hjorth-Hansen H., Olsson B., Wadenvik H., Sundan A., Standal T. (2012). Imatinib inhibits proliferation of human mesenchymal stem cells and promotes early but not late osteoblast differentiation in vitro. J. Bone Miner. Metab..

[39] Hock J.M., Krishnan V., Onyia J.E., Bidwell J.P., Milas J., Stanislaus D. (2001). Osteoblast apoptosis and bone turnover. J. Bone Miner. Res. Off. J. Am. Soc. Bone Miner. Res..

[40] Jilka R.L., Weinstein R.S., Bellido T., Roberson P., Parfitt A.M., Manolagas S.C. (1999). Increased bone formation by prevention of osteoblast apoptosis with parathyroid hormone. J. Clin. Investig..

[41] Huang H.T., Cheng T.L., Lin S.Y., Ho C.J., Chyu J.Y., Yang R.S., Chen C.H., Shen C.L. (2020). Osteoprotective roles of green tea catechins. Antioxidants.

[42] Mackie E.J., Ahmed Y.A., Tatarczuch L., Chen K.S., Mirams M. (2008). Endochondral ossification: How cartilage is converted into bone in the developing skeleton. Int. J. Biochem. Cell Biol..

[43] Sims N.A., Martin T.J. (2015). Coupling signals between the osteoclast and osteoblast: How are messages transmitted between these temporary visitors to the bone surface?. Front. Endocrinol..

[44] Li C., Fennessy P. (2021). The periosteum: A simple tissue with many faces, with special reference to the antler-lineage periostea. Biol. Direct.

[45] Zarei A., Ballard A., Cox L., Bayguinov P., Harris T., Davis J.L., Roper P., Fitzpatrick J., Faccio R., Veis D.J. (2021). Osteolineage depletion of mitofusin2 enhances cortical bone formation in female mice. Bone.

[46] Taylor R.C., Cullen S.P., Martin S.J. (2008). Apoptosis: Controlled demolition at the cellular level. Nat. Rev. Mol. Cell Biol..

[47] He C., Klionsky D.J. (2009). Regulation mechanisms and signaling pathways of autophagy. Annu. Rev. Genet..

[48] Jiang A., Guo H., Wu W., Liu H. (2021). The crosstalk between autophagy and apoptosis is necessary for myogenic differentiation. J. Agric. Food Chem..

[49] Wang N., Xu P., Wu R., Wang X., Wang Y., Shou D., Zhang Y. (2021). Timosaponin BII improved osteoporosis caused by hyperglycemia through promoting autophagy of osteoblasts via suppressing the mTOR/NFκB signaling pathway. Free. Radic. Biol. Med..

[50] Li D.Y., Yu J.C., Xiao L., Miao W., Ji K., Wang S.C., Geng Y.X. (2017). Autophagy attenuates the oxidative stress-induced apoptosis of Mc3T3-E1 osteoblasts. Eur. Rev. Med. Pharmacol. Sci..

